# Designing of a new transdermal antibiotic delivery polymeric membrane modified by functionalized SBA-15 mesoporous filler

**DOI:** 10.1038/s41598-024-60727-x

**Published:** 2024-05-06

**Authors:** Mahya Samari, Soheila Kashanian, Sirus Zinadini, Hossein Derakhshankhah

**Affiliations:** 1 Department of Applied Chemistry, Faculty of Chemistry, Razi University, Kermanshah, Iran; 2 Nanobiotechnology Department, Faculty of Innovative Science and Technology, Razi University, Kermanshah, Iran; 3 Environmental Research Center (ERC), Razi University, Kermanshah, Iran; 4 Pharmaceutical Sciences Research Center, Health Institute, Kermanshah University of Medical Sciences, Kermanshah, Iran; 5 USERN Office, Kermanshah University of Medical Sciences, Kermanshah, Iran

**Keywords:** Transdermal drug delivery, Mesoporous filler, Polymeric membrane, SBA-15, Glutamine, Azithromycin, Biotechnology, Molecular medicine

## Abstract

A new drug delivery system using an asymmetric polyethersulfone (PES) membrane modified by SBA-15 and glutamine-modified SBA-15 (SBA-Q) was prepared in this study by the aim of azithromycin delivery enhancement in both in vitro and ex vivo experiments. The research focused on optimizing membrane performance by adjusting critical parameters including drug concentration, membrane thickness, modifier percentage, polymer percentage, and pore maker percentage. To characterize the fabricated membranes, various techniques were employed, including scanning electron microscopy, water contact angle, and tensile strength assessments. Following optimization, membrane composition of 17% PES, 2% polyvinylpyrrolidone, 1% SBA-15, and 0.5% SBA-Q emerged as the most effective. The optimized membranes demonstrated a substantial increase in drug release (906 mg/L) compared to the unmodified membrane (440 mg/L). The unique membrane structure, with a dense top layer facilitating sustained drug release and a porous sub-layer acting as a drug reservoir, contributed to this improvement. Biocompatibility assessments, antibacterial activity analysis, blood compatibility tests, and post-diffusion tissue integrity evaluations confirmed the promising biocompatibility of the optimized membranes. Moreover, long-term performance evaluations involving ten repeated usages underscored the reusability of the optimized membrane, highlighting its potential for sustained and reliable drug delivery applications.

## Introduction

Sustained released drug delivery in pharmacotherapy is frequently imperative for effectively managing underlying diseases. Such pharmacotherapy encompasses the administration of maintenance medications, which play a pivotal role in symptom control, complication prevention, and overall enhancement of health outcomes^1–3^. However, long-term drug use can also carry potential risks and challenges, including poor absorption^4,5^, first-pass metabolism^6,7^, dosage limitations^8,9^, adherence^10,11^, storage and stability^12,13^, cost^14^, and side effects^15^, which healthcare professionals should carefully monitor and manage. To overcome the challenges ahead, various routes of drug delivery have been identified, including the oral route^16^, inhalation route^17^, intravenous route^18^, subcutaneous route^19^, and transdermal route^20^. Various routes of transdermal delivery are common including:

1. Transdermal Patches^21^:
  Advantages:
    Convenient and non-invasive method of drug delivery.
    Offers sustained release of medication, maintaining constant drug levels in the bloodstream.
    Avoids first-pass metabolism, leading to improved bioavailability.
    Enhances patient compliance due to ease of use and long-lasting effects^22^.
  Disadvantages:
    Limited to drugs with specific physicochemical properties that allow for adequate skin permeation.
    Slow onset of action compared to other routes of administration.
    Potential skin irritation or allergic reactions at the patch site.
2. Transdermal gels and creams^23^:
  Advantages:
    Offers flexibility in drug formulation and dosage adjustments.
    Rapid absorption through the skin, leading to quicker onset of action.
    Can be applied to a larger surface area, allowing for higher drug doses.
    Can be easily discontinued if adverse reactions occur.
  Disadvantages:
    Requires careful application to ensure proper drug absorption.
    May leave a residue on the skin or cause skin greasiness.
    Some drugs may not adequately penetrate the skin barrier, limiting their effectiveness.
    Potential for accidental transfer to others, especially in the case of topical gels.
3. Iontophoresis^24^:
  Advantages:
    Enhances transdermal drug delivery through the application of a low electrical current, facilitating drug penetration through the skin.
    Allows for the delivery of charged molecules that may not otherwise permeate the skin barrier effectively.
    Can be used to target specific areas of the body for localized drug delivery.
    Non-invasive and generally well-tolerated.
  Disadvantages:
    Requires specialized equipment and expertise for administration.
    Potential for skin irritation or burns at the site of electrode placement.
    Limited to drugs that are amenable to iontophoretic delivery.
    Treatment duration may be longer compared to other transdermal methods.
4. Microneedle systems^25^:
  Advantages:
    Enhances drug penetration by creating micropores in the skin, bypassing the stratum corneum barrier.
    Allows for controlled and precise drug delivery, minimizing systemic side effects.
    Can be used for a wide range of drugs, including those with poor skin permeability.
    Promotes rapid onset of action and dose optimization.
  Disadvantages:
    Requires specialized equipment for application, potentially increasing costs.
    Risk of skin irritation, infection, or injury if not administered properly.
    Limited to drugs that are stable in the presence of microneedle insertion.
    Potential for variability in drug delivery depending on skin thickness and individual skin characteristics.

In this case, transdermal patches (TPs) represent a sophisticated medication delivery system that facilitates the passage of drugs through the skin and subsequent entry into the bloodstream^21^. This approach has found extensive utility across diverse therapeutic domains, including effective drug administration, hormonal replacement therapy, and nicotine cessation interventions^26^. Compared to traditional oral medications, TPs offer several advantages, such as a more consistent and controlled release of drugs, lower risk of side effects, and improved patient compliance^27^. However, the efficacy of TPs can be varied by factors such as patch design and the properties of the drug by itself.

Polyethersulfone (PES) is a thermoplastic polymer renowned for facilitating the development of TPs and adhesive devices for transdermal drug delivery. In this essence, PES has been dubbed as a superior polymer choice in this application owing to its exceptional physical and chemical attributes, including remarkable high-temperature resistance, commendable biocompatibility, and outstanding dimensional stability^28,29^. The PES is employed as a backing layer or support material within TPs, serving the dual purposes of endowing mechanical strength and safeguarding the active ingredients within the patch. Polyurethane and ethylene–vinyl acetate copolymers are other frequently utilized materials in the composition of TPs^30^. Still, PES has become a popular polymer due to its favorable properties and ability to maintain its mechanical strength and barrier properties over time.

SBA-15, a mesoporous silica material renowned for its ordered hexagonal array of cylindrical mesoporous, offers many opportunities in materials science and nanotechnology^31^. Synthesized through templating methods, wherein a surfactant molecule acts as a template around which the silica precursor polymerizes and subsequent removal of the surfactant template leaves behind the ordered mesoporous structure, SBA-15 exhibits exceptional properties^32^. Moreover, its tunable pore size and impressive thermal and chemical stability contribute to its versatility, making it suitable for a broad spectrum of applications ranging from drug delivery and sensing to catalysis and separation processes. SBA-15's biocompatibility, tunable pore size, and controlled drug release kinetics further position it as a promising candidate in drug delivery^33^.

Glutamine is an amino acid with a side chain containing amino and carboxyl groups, making it amenable to covalent bonding or adsorption onto the silica surface^34^. The modification process typically begins with activating the silica surface, followed by introducing glutamine molecules. This modification imparts specific functionalities to the SBA-15 material, such as enhanced biocompatibility, targeted drug delivery, or improved adsorption properties and hydrophilicity. Glutamine-modified SBA-15 materials have shown promise in biomedical applications due to their ability to interact with biological molecules and cells.

The present study has been focused on developing a drug delivery system (DDS) using asymmetric porous membranes as a proper and safe route for drug delivery. This approach involves releasing the drugs from the pores of the membrane via a diffusion-based release. A dense membrane top layer is incorporated into the design to enhance further drug delivery efficiency, which prevents initial burst release and enables long-term drug release.

Several parameters, such as thickness (150, 300, 450, and 600 µm), drug concentration (500, 1000, and 1500 mg/L), and modifier percentage (0.5, 1, 1.5, and 2% of SBA-15 and SBA-15 modified by glutamine (SBA-Q)), were optimized to achieve the desired DDS performance. The release profile of the azithromycin (AZI) drug was investigated in simulated human blood fluid for 24 h, and the drug release profiles were also assessed in vitro. This study provides a promising approach for developing an effective and safe DDS using asymmetric porous membranes. The results demonstrate the potential of this approach to be used in various biomedical applications.

## Materials and methods

### Materials

Different chemicals were used to fabricate the membranes and to synthesize mesoporous SBA-15 and SBA-Q as membrane modifiers. Polyethersulfone (PES; MW: 58,000 g/mol) was prepared from BASF Co., Germany. Hydrochloric acid (HCl 37%), tetraethoxysilane (TEOS 98%), glutamine, (3-chloropropyl) trimethoxysilane (CPTMS), toluene, and dimethylacetamide (DMAc) were all purchased from Merck Co. Germany. Pluronic P123 ((EO)_20_(PO)_70_(EO)_20_, MW: 5800) was supplied from Sigma-Aldrich Co. USA. Phosphate buffered saline (PBS) tablets were obtained from Zist Mavad Pharmed Co. Iran. All the reactants were used as received with no further purification. In all examinations, distilled water was applied.

### Synthesis of SBA-15

The synthesis of SBA-15 involved a series of controlled conditions. Initially, a mixture of P123 (4 g) and HCl (120 mL, 2 M) and distilled water (45 mL) was prepared. Subsequently TEOS, (9 mL) was introduced to the mixture while continuous stirring for 24 h. After stirring, the mixture was subjected to a heat treatment at 100 ℃ for 24 h. Subsequently, the resultant product was washed with distilled water, followed by filtration and drying^35^.

### SBA modification

In this part of experimental procedure, SBA-15 (1 g) was dissolved in toluene (50 mL), then CPTMS (1 mL) was added to the resulting solution. The obtained mixture was then subjected to reflux at a temperature of 110 °C. After 24 h, the solution was dried to form chlorinated SBA material. Subsequently, chlorinated SBA (1 g) and glutamine (1.2 g) were combined in a 50 mL toluene flask. The resulting mixture was then subjected to reflux at the same temperature of 110 °C for 24 h. After completing this reaction, the resulting substance was carefully dried, leading to the functionalization of SBA with the amino acid, thereby generating SBA-Q.

### Membrane fabrication

Flat-sheet polymeric membranes were fabricated by implementing phase inversion methods.

This method is a commonly used technique for fabricating asymmetric membranes^36^. In this case, SBA-15 and SBA-Q were employed as membrane modifiers, contributing to their enhanced properties in hydrophilicity and membrane structure. The composition of the membrane casting solutions was determined based on the data presented in Table 1. In brief, various quantities of SBA and SBA-Q were dispersed in DMAc, and to sonicated to ensure proper dispersion. Subsequently, the primary polymer, PES, and PVP, were introduced into the mixture, while continuous stirring for 24 h. This process was aimed to achieve homogeneous mixtures with consistent properties.

**Table 1:** The composition of the casting solutions.

Membrane code	PES, wt%	PVP, wt%	DMAc, wt%	NPs, wt%
M1	17	2	81	0
M2	17	2	80.5	0.5*a*
M3	17	2	80	1*a*
M4	17	2	79.5	1.5*a*
M5	17	2	79	2*a*
M6	17	2	80.5	0.5*b*
M7	17	2	80	1*b*
M8	17	2	79.5	1.5*b*
M9	17	2	79	2*b*

*PES* polyethersulfone, *PVP* polyvinylpyrrolidone, *DMAc* dimethylacetamide.

SBA-15 (a), SBA-Q (b).

To improve the uniformity of the solutions and eliminate any entrapped air bubbles, each solution underwent sonication at room temperature for 20 min. During the membrane fabrication, the solution was applied onto pristine glassy plates using a film applicator in the desired thickness (150, 300, 450, and 600 µm). The plate was then immersed in a non-solvent bath containing distilled water, which used the formation of flat-sheet polymeric membranes. To ensure the completion of the phase separation process, the fabricated membranes were immersed in fresh distilled water overnight.

In this experimental study, different membranes were fabricated and examined to obtain the optimum percentage of polymer and pore maker agent (13, 17, and 21% of PES with 0, 2, and 4% PVP). Next, the membrane with the best performance (17% PES and 2% PVP) was chosen as the unmodified membrane composition and then modified by a proper nanoparticle as the modifier.

### Drug loading

In this study, the non-degradable polymer has been used to design asymmetric flat-sheet membranes for multiple uses. After fabricating the membranes, the drug solution (500, 1000, and 1500 mg/L) was loaded from the membrane sub-layer, which has larger pores, to load more amounts of the drug. Also, this method prevents the surface absorption of the drug by the membrane surface and explosive release at the initial release time. For this case, a manufactured Plexiglas setup with continuous stirring was designed to expose the sub-layer of the membranes to the drug solution.

### In vitro drug release

Drug release from the prepared membranes was investigated utilizing a Franz diffusion cell with a membrane surface area of 12.56 cm^2^.The system operated under gentle continuous stirring at 100 rpm. In summary, the upper layer of the fabricated membranes, serving as the controlling layer, remained fixed while being exposed to PBS at pH 7.4. Throughout the experiments, 1 mL of the released medium was withdrawn at designated time intervals (1 h), and an equal volume of fresh PBS was replaced. The drug release was monitored from the smaller pores engineered within the top layer of the membrane, facilitating continuous and controlled release. The concentration of the released drug (AZI) in the medium was quantified using a UV–Vis spectrophotometer at a wavelength of 208 nm.

### Drug loading and entrapment efficiency

The drug loading (DL%) and entrapment efficiency (EE%) of the fabricated membranes were evaluated by using an indirect method^37^. The EE% and DL% of the drug within the fabricated membranes can be calculated according to Eqs. (1) and (2), respectively.

1 $$\text{EE\%}=\left[\frac{\text{Total amount of drug added}-\text{Amount of drug in the aqueous phase}}{\text{Total amount of drug added}}\right]\times 100,$$

2 $$\text{DL\%}=\left[\frac{\text{Amount of drug entrapped in the membrane}}{\text{Total weight of membrane}}\right]\times 100,$$

### Characterization techniques

The examination of the membrane cross-section morphology was conducted employing a scanning electron microscope (SEM; TESCAN MIRA III). Small sections of the membrane underwent immersion in liquid nitrogen for approximately 70–90 s, followed by fracturing of the frozen membrane pieces and subsequent drying. To ensure adequate conductivity, the samples were coated with a layer of gold. Subsequently, the SEM images were captured under highly vacuumed conditions with a 20 kV acceleration voltage.

Fourier transform infrared spectroscopy (FT-IR) analysis (Bruker alpha spectrometer, Germany) was employed to investigate the chemical bonds of the molecules.

To assess the hydrophilicity of the membrane, water contact angle (WCA) measurements were performed. This method provides insight into the surface propensity for water spreading. A droplet of distilled water (4 µL) was carefully deposited onto the membrane surface. After 15 s, the angle formed between the water droplet and the membrane surface was recorded using a digital microscope (contact angle meter XCA-50). To enhance reliability, measurements were taken at five distinct random locations on each sample, and the average data were subsequently reported^36^. The mechanical properties of the fabricated membranes were evaluated based on a tensile quality approach, by using a uniaxial malleable device (STMctrlr) at room temperature and speed of 500 mm/min^38^.

### Water vapor transition rate

The water vapor transition rate (WVTR) of the fabricated membranes was examined according to the evaporated water mass (24 h, 5 mL) from dressings-capped bottles^39^.

### Blood compatibility assay

Blood obtained from a healthy volunteer was utilized to assess the hemocompatibility of the fabricated membranes. Fresh anticoagulated blood was combined with PBS in a 1:1.5 ratio, followed by treatment at 37 °C for 1 h. Positive and negative controls were established using diluted blood with deionized water (resulting in 100% lysis) and PBS (resulting in 0% lysis), respectively. After the incubation period, the samples were centrifuged at 1500 rpm for 10 min. Subsequently, the absorption of released hemoglobin was measured at 545 nm using a Microplate Reader^40^.

### Antibacterial tests

The agar disk diffusion method was investigated for the antibacterial activity of the fabricated membranes. Mueller–Hinton agar is commonly used as the growth medium. The agar was poured into petri dishes and allowed to solidify. Then a standardized inoculum of the test microorganism was spread evenly over the surface of the agar using a sterile swab. The samples were placed onto the agar surface using sterile forceps. The agar plates are then incubated at room temperature for the growth of the test microorganism for 24 h. After incubation, the plates were examined for the presence of zones of inhibition around the samples. The diameter of each zone is measured using a calibrated ruler.

In this study, four different Gram-positive (*Streptococcus sobrinus* and *Staphylococcus aureus*) and Gram-negative bacteria (*Pseudomonas aeruginosa* and *Escherichia coli*) were applied.

### Cytotoxicity assessment (MTT assay)

Cell viability and membrane integrity were assessed using the 3-(4,5-dimethylthiazol-2-yl)-2,5-diphenyltetrazolium bromide (MTT), a widely employed in vitro cytotoxicity evaluation method^41^. This assay relies on the activity of mitochondrial dehydrogenases to indicate cellular viability. Functioning mitochondria in live cells reduce the MTT reagent to form insoluble formazan crystals. Following incubation, the formazan crystals are solubilized, and the absorbance of the resulting solution is measured at 570 nm using a spectrophotometer. This measurement quantitatively assesses cell viability, with higher absorbance values indicating greater metabolic activity and viability.

### Ex vivo experiments

The ex vivo experiments conducted using skin samples from healthy mice to investigate how effectively our modified membrane could penetrate. Before commencing the experiments, our university’s Animal Ethics Committee thoroughly reviewed and approved the study (IR.KUMS.AEC.1400.014, Research Ethics Committees of Laboratory Animals—Kermanshah University of Medical Sciences). We utilized male Medical Research Institute mice (8 weeks, ~ 30 g), ensuring they were housed under standard conditions conducive to their well-being.

The mice were sacrificed under deep anesthesia induced by intraperitoneal injection of ketamine (80 mg/kg)/Diazepam (0.5 mg/kg) cocktail. Bare and optimized modified membranes were employed to evaluate the ex vivo permeation characteristics of mouse skin. Before the commencement of the study, thorough shaving of hair and removal of subcutaneous fat tissues were carried out. The presented experimental study has been reported in accordance with ARRIVE guidelines. Also, all methods were performed in accordance with the relevant guidelines and regulations.

### Statistical analysis

The results were represented as mean ± standard error of the mean (SEM). All statistical analyses were submitted to SPSS *ver*. 23.0 statistical software (SPSS Inc., Chicago, IL, USA). The comparison of means for data was performed using one-way analysis of variance (ANOVA) and post hoc least significant difference (LSD) test. The P value ≤ 0.05 was considered as statistically significant level.

## Result and discussion

### Membrane cross-sectional SEM images

**Figure 1:**
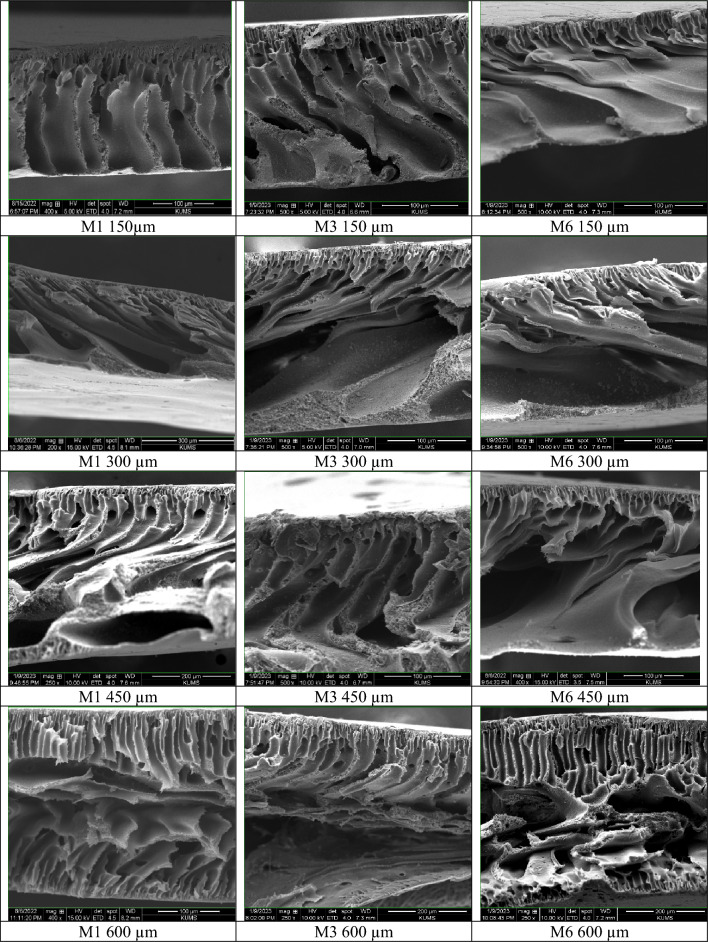
The cross-sectional scanning electron microscopy image of the M1, M3, and M6 membranes in different thickness (150, 300, 450, and 600 µm).

The cross-sectional composition of the fabricated membranes was examined through SEM analysis to investigate the influence of membrane modification on morphological attributions. Four distinct thicknesses were evaluated; the findings are visually presented in (Fig. 1). The obtained data shows that the membranes exhibited a structural arrangement, including a porous finger-shaped sub-layer and a compact top layer. It was observed that the modified membranes exhibited larger and more regular channels, a phenomenon that aligns with the principles delineated in the Hagen-Poiseuille relationship^36^.

An unexpected and distinct revelation occurred during the phase inversion process of 600 μm thick membranes. An interesting phenomenon occurred: water permeated both sides of the membrane and formed two compact top layers. The least difference in structure was observed among the M1600, M3600 and M6600 membranes. However, the M1150 membranes showed an interesting feature: the sublayer showed the absence of detectable pores, indicating solid adhesion and entanglement of the polymer strands. In contrast, the M3450 and M6450 membranes, which were modified with mesoporous filler dispersed among the polymer filaments, formed hydrogen bonds that prevented the entanglement of the primary filaments.

Consequently, this led to the generation of pores and increased porosity during the phase inversion process. Both kinetic and thermodynamic factors control the complex dynamics of the phase inversion process. Notably, the latter has a more significant effect on modified membranes and forms uniform and regular channels.

Increasing the thickness of the membrane created a more precise cavity under the layer that acts as a storage or reservoir pore. However, when the thickness exceeded a certain threshold, the decrease in kinetic coefficient caused the polymer filaments to return to their original stiffness, resulting in smaller holes. This phenomenon was especially evident in the case of 600 μm thick membranes. Clusters within the modified membranes indicated the incorporation of mesoporous filler loaded into the membrane structure.

These observations showed that the modified membranes (M3 and M6) showed significant pores in the sub-layers, potentially suitable for storing pharmaceutical solutions. Smaller holes in the top layer are designed for an extended, controlled release. Modification of the membranes resulted in a more uniform and porous structure, which offered promising applications in DDSs.

### Mechanical properties

**Figure 2:**
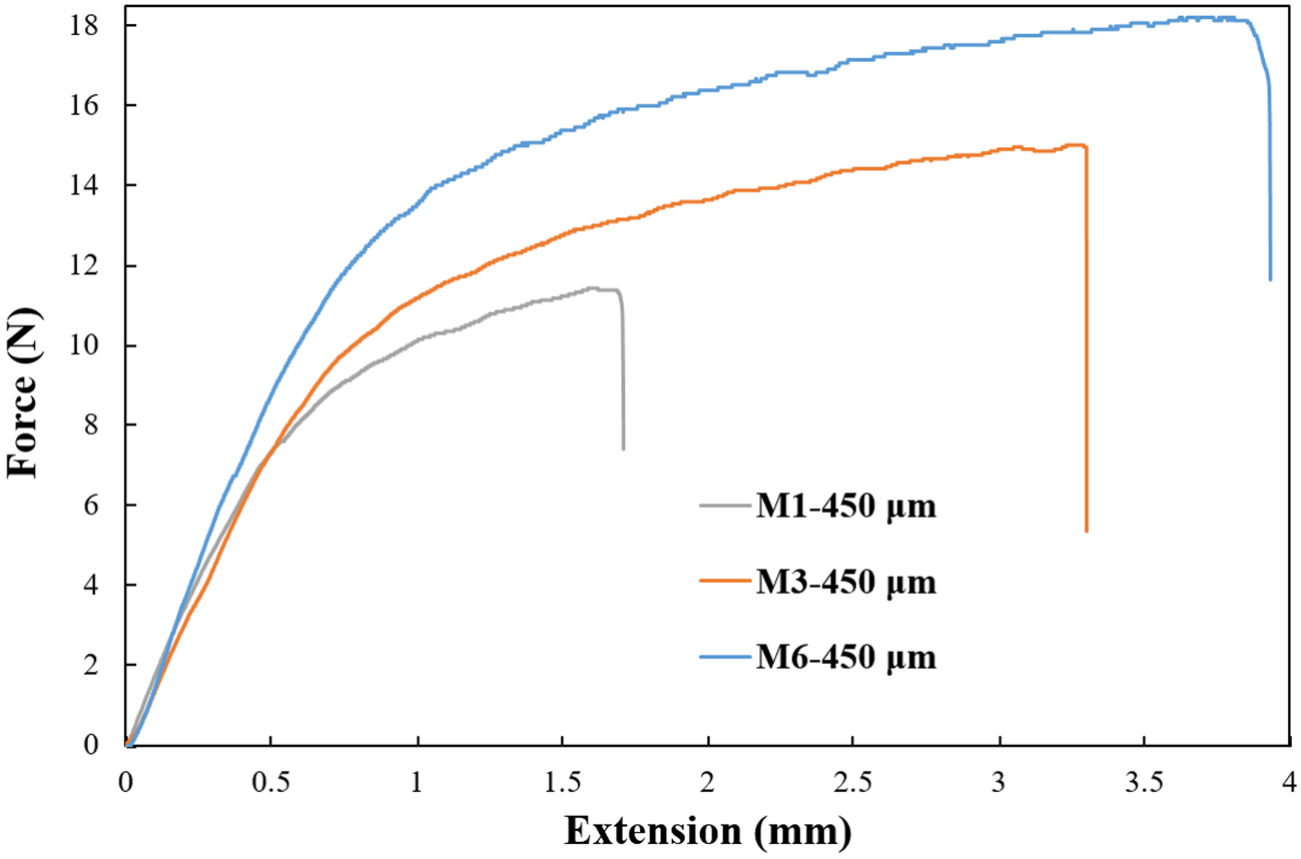
The tensile strength evaluation of the fabricated M1 and M3, and M6 membranes in optimal membrane thicknesses (450 µm).

The mechanical properties of the fabricated membranes were evaluated utilizing the tensile strength methodology, which provided crucial insights into their performance when subjected to mechanical stress^42^. This mechanical strength and flexibility enhancement was observed after incorporating NPs into the membrane structure (Fig. 2). The obtained findings emphasize the statistical significance of the observed differences in mechanical properties, indicating the reliability of the results. This finding suggests that the addition of SBA-15 and SBA-Q positively impacts the mechanical properties of the membranes, potentially making them more durable and flexible. This could have significant implications for applications where such membranes are used, such as in filtration processes, separation technologies, or biomedical devices.

Forati et al*.* reported that adding Graphene oxide into PES nanocomposites notably enhanced PES films’ mechanical properties and thermal stability^43^. Feng Li and coworkers also reported that loading TiO_2_ NPs in PES texture improved the membrane mechanical strength^44^.

### Wettability

A low water contact angle (WCA) indicates that the surface is more hydrophilic, meaning it has a higher affinity for water, whereas a high WCA suggests the surface is more hydrophobic, repelling water. The high WCA of the bare membrane indicates its hydrophobic nature, meaning water droplets bead up and do not spread out on its surface due to the low surface energy of the membrane material. This characteristic effectively prevents the wetting of the water surface by the membrane. A bare PES-based flat-sheet membrane inherently possesses hydrophobic characteristics according to its high WCA^45^. However, through a modification process involving the incorporation of hydrophilic SBA-15 and SBA-Q into the membrane matrix, the surface properties of the PES-based membrane can be altered. By incorporating SBA-15 into the membrane matrix, the surface properties of the membrane change, leading to a decrease in the WCA. In the case mentioned, the WCA decreased from 69.24° to 51.84° for the M3 (Fig. S3). This decrease in water contact angle indicates that the modified membrane becomes less hydrophobic and more hydrophilic after incorporating SBA-15. This modification process can be advantageous in various applications.

In addition, incorporating SBA-Q further increased the hydrophilicity, as evidenced by a decrease in the WCA of the membranes. In particular, for the modified M6 membrane, the WCA decreased to 42.26°, indicating a significant improvement in the wetting behavior. The enhanced hydrophilicity observed in SBA-Q can be attributed to the presence of polar amide groups in its side chain, which facilitated the formation of hydrogen bonds with water and other polar molecules. This feature was crucial in the function and structure of proteins containing glutamine residues.

These results, which the membrane hydrophilicity enhances by loading hydrophilic additives to the texture, were consistent with the results reported in the literature^36,46^.

### Drug loading and entrapment efficiency

Figure S7 demonstrates comparable drug loading rates and entrapment efficiencies within the membrane matrix. The concentration of the drug solution directly influences the drug loading rate. Increasing the drug concentration notably enhanced the loading content. The optimally modified membrane (M6) exhibited an entrapment efficiency of 74.3% and drug loading of 38.1%.

### Drug release and long-term performance

The AZI is an antibiotic commonly used to treat various bacterial infections, including skin infections^47^. This part of the study investigated the release characteristics of loaded AZI from the fabricated membranes. The primary objective was determining the ideal filler concentration to achieve maximum storage volume and efficient membrane modification.

There are several limitations associated with the use of degradable polymers in TPs, for which non-degradable polymers are preferred. These limitations include limited stability, variable degradation rates, difficulty in controlling drug release, and poor loading capacity^48,49^. Therefore, experiments were conducted to investigate the performance of M3 and M6 membranes in long-term drug delivery under optimal conditions (450 μm, 1000 mg/L). To ensure the integrity and cleanliness of the membranes during the long evaluation period, a dual membrane system was used that allowed regeneration and purification of the membranes. After an initial evaluation of membrane performance, an autoclave process was used to remove any potential contaminants. Then the membranes were immersed in distilled water for 30 min. This thorough cleaning method ensures the removal of impurities and thus facilitates the reusability of the membranes real world applications.

**Figure 3:**
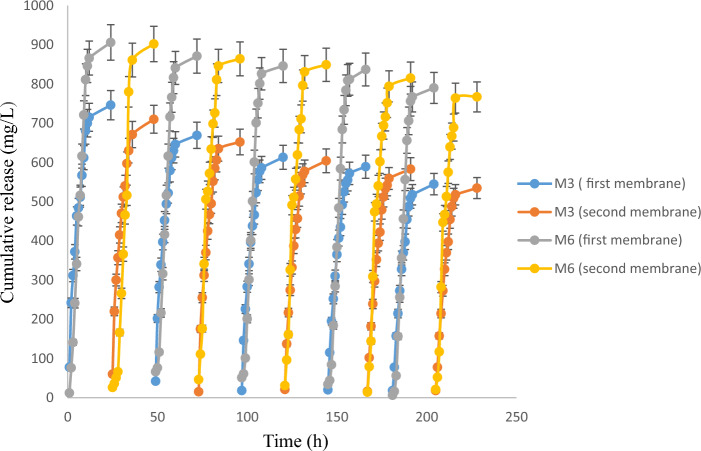
The long-term performance of the M3, and M6 in optimal operational conditions (1000 mg/L, 450 µm) in ten frequent cycles of drug release (n = 3).

By adopting this approach, the trial run achieved affordability and sustainability. Simultaneously, while the first membrane underwent recovery and cleaning, a fresh membrane was introduced into the Franz cell to start the second evaluation cycle. This alternating pattern was repeated for ten cycles (Fig. 3), each occurring consecutively. This complete rotation ensured continuous and uninterrupted evaluation of membrane performance throughout the experiment.

### Water vapor transition rate

The evaluation of vapor transmission through TPs holds significant importance in determining their suitability as wound dressings. Achieving an optimal WVTR is essential to maintain the ideal moisture balance in the affected area. Excessive WVTR could lead to undesirable skin or wound dehydration, whereas insufficient transmission might hinder the healing process by trapping exudate^50^. Therefore, TPS must exhibit an optimal WVTR value to ensure effective wound management and promote proper healing.

The utilization of SBA-15 as a modifier for PES membranes presents a promising strategy for enhancing their WVTR, as demonstrated by the increase from 2.55 for M1 to 5.95 for M3. This modification effectively strengthens the membrane's surface and creates numerous channels to facilitate the transport of water vapor across the membrane. Furthermore, incorporating SBA-Q results in a PES membrane with a more porous and open structure, thereby increasing its hydrophilicity and significantly enhancing the WVTR. This is evidenced by the notable increase from 2.55 for M1 to 13.6 for M6.

### Cell cytotoxicity

Cell viability is a critical parameter for assessing the health and functionality of cells when exposed to various assaults determined through the utilization of the MTT assay, which evaluates mitochondrial activity^51^. In the current investigation, the cell viability of the optimized TPs, particularly M1, M3, and M6, was evaluated at 24-, 48-, and 72 h intervals and compared to a control group displaying acceptable (~ 80%) cell viability (Fig. S8).

The results obtained are significant in assessing the potential cytotoxic impacts of TPs and ensuring their safety profile. By examining cell viability in response to exposure to the fabricated TPs at specified time points, valuable insights into their biocompatibility and absence of harmful cytotoxic effects can be gleaned. These findings serve as a pivotal foundation for evaluating the viability and appropriateness of TPs as DDSs, bolstering confidence in their safety profile and streamlining their successful incorporation into clinical applications.

### Hemo-compatibility

As fabricated membranes are intended to be in contact with the wound site, ensuring blood compatibility is critical. PES-based TPs, widely recognized for their homo-compatibility, are generally well tolerated by the human body and do not induce an immune response or inflammation^52^. The extent of hemolysis induced by the membranes was quantified to assess the hemocompatibility of the bare (M1 = 6.84%) and the optimally modified membranes (M3 = 5.84 and M6 = 4.77%).

The findings showed that the modified membranes indicated significantly lower hemolytic levels than the positive control, where red blood cells were exposed to distilled water, resulting in osmolysis^53^. These observations indicate a favorable compatibility of the membranes, as indicated by their minimal effect on red blood cells following the hydrophilicity enhancement. The measured hemolytic levels confirm the biocompatibility of PES-based TPs and strengthen their potential for safe and effective use in clinical settings. These results provide insight into the blood compatibility of the modified membranes and confirm their suitability for contact with body fluids, confirming their potential as a viable option for wound management.

### Kinetic evaluations

Mathematical models are commonly used to describe the release characteristics of drugs from delivery systems, elucidating the relationship between the amount of drug released and the elapsed time. Four widely utilized mathematical models for drug release analysis include the zero-order, first-order, Higuchi, and Korsmeyer–Peppas models^54^.

In order to select the most suitable model for a specific DDSs, it is imperative to thoroughly assess the fit of each model to experimental data. This evaluation involves comparing the R-squared values of each model, as outlined in Table 2. Upon analysis, it is evident that the zero-order model emerges as the most appropriate mathematical model for depicting the release characteristics of the desired drug. The meticulous selection of the optimal model, guided by the scrutiny of R-squared values, ensures an accurate depiction of drug release kinetics and provides insights into the underlying release mechanism. By leveraging the most fitting mathematical model, researchers can adeptly design and optimize DDSs, enabling precise control over drug release kinetics and ultimately augmenting therapeutic outcomes.

**Table 2:** The kinetic evaluation of the bare and optimally modified membranes in four mathematical models.

	Zero-order	First order	Higuchi	Korsmeyer–Peppas
Equation	*Q*_*t*_ = *Q*_0_ + *K*_0_*t*	log* Q*_*t*_ = *logQ*_0_* − K*_*t*_*/*2.303	*Q* = *K*_*H*_ ×* t*^*1/2*^	log *(M*_*t*_*/M*_*∞*_*)* = log*k* + *n*log*t*
M1	K_0_ = 28.476 R^2^ = 0.7064	K_1_ = − 0.2143 R^2^ = 0.375	K_H_ = 83.762 R^2^ = 0.9253	K_R_ = 76.24 n = 0.66 R^2^ = 0.6669
M3	K_0_ = 26.607 R^2^ = 0.973	K_1_ = − 0.3823 R^2^ = 0.9204	K_H_ = 67.505 R^2^ = 0.9482	K_R_ = 8.8 n = 36.04 R^2^ = 0.8918
M6	K_0_ = 49.375 R^2^ = 0.9966	K_1_ = − 0.3698 R^2^ = 0.8168	K_H_ = 120.85 R^2^ = 0.9038	K_R_ = 8.8 n = 36.04 R^2^ = 0.9458

M1: 17% polyethersulfone and 2% polyvinylpyrrolidone, M3: 17% polyethersulfone, 2% polyvinylpyrrolidone and 1% SBA-15, and M6: 17% polyethersulfone, 2% polyvinylpyrrolidone, and 0.5% SBA-15 modified by glutamine.

### Antibacterial assay

**Figure 4:**
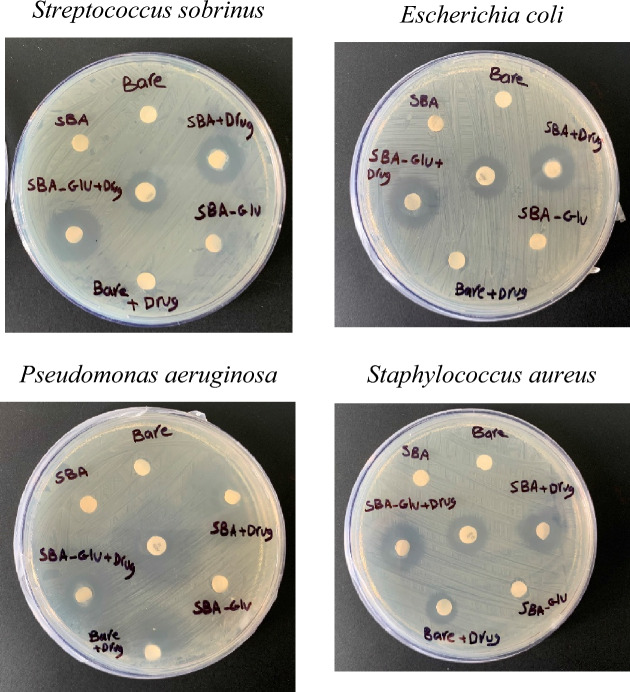
Antibacterial evaluation of the M1 (unmodified, 17% PES), M3, and M6 (optimally modified membranes), with and without drug loading (1000 mg/L, 450 µm).

The agar disk diffusion method is suitable for assessing the antibacterial efficacy of TPs, as it emulates the release of active substances from the patch into the skin^55^. The results demonstrated that the fabricated membranes, with drug loading (M1, M3, and M6), exhibit enhanced antibacterial activity compared to the control group (Fig. 4, Table 3). This increase in antibacterial efficacy can be attributed to the appropriate drug release profile achieved by the modified membranes. The presence of the drug within the membranes contributes to their ability to effectively inhibit bacterial growth, leading to promising antibacterial activity.

**Table 3:** The inhibitory effect of the antibiotic-treated disk against pathogens.

Pathogens	Diameter of inhibition zone (mm)						
	M3 + drug	M6 + drug	M6	M3	M1	M1 + drug	Control
*S. sobrinus*	17.0 ± 1.0^b^	21.3 ± 0.6^a^	0.0 ± 0.0^d^	0.0 ± 0.0^d^	0.0 ± 0.0^d^	0.0 ± 0.0^d^	13.7 ± 0.6^c^
*P.aeruginosa*	30.7 ± 0.6^b^	33.0 ± 1.0^a^	0.0 ± 0.0^e^	0.0 ± 0.0^e^	0.0 ± 0.0^e^	21.3 ± 1.5^d^	27.7 ± 0.6^c^
*S. aureus*	19.0 ± 1.0^a^	19.7 ± 1.5^a^	0.0 ± 0.0^d^	0.0 ± 0.0^d^	0.0 ± 0.0^d^	11.0 ± 1.0^c^	15.7 ± 0.6^b^
*E. coli*	17.7 ± 1.5^b^	19.3 ± 1.1^a^	0.0 ± 0.0^d^	0.0 ± 0.0^d^	0.0 ± 0.0^d^	0.0 ± 0.0^d^	15.3 ± 0.6^c^

Values are mean ± standard error of triplicates.

^a–e^Means in the same row with different lowercase letters differed significantly (p < 0.05).

These findings underscore the importance of optimizing the drug release profile within TPs to maximize their antibacterial effectiveness.

### Ex vivo experiments

**Figure 5:**
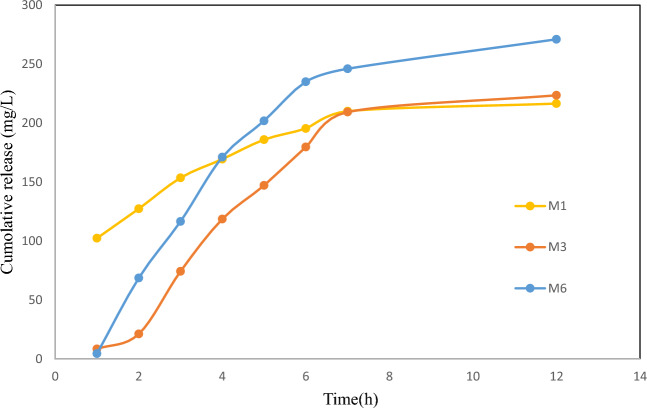
Ex vivo comparative evaluation of drug release for the M1, M3 and M6 in optimal azithromycin solution concentrations (1000 mg/L) and membrane thicknesses (450 µm).

In this phase of the study, a rigorous investigation was aimed at evaluating the drug release performance of two distinct membranes, denoted as M1 and M3, employing authentic samples derived from the dorsal skin of mice. A fine preparation process was employed for the skin samples, which were subsequently placed in Franz cell, ensuring secure and optimized attachment of the membranes. The resulting data, presented graphically in Fig. 5, facilitated a clear and comprehensive comparison of the outcomes.

Significantly, the M1 membrane exhibited a conspicuous pattern characterized by abrupt and intermittent drug release, whereas the M3 and M6 membranes demonstrated a consistent and sustained release profile. The onset of drug release for all membranes occurred approximately one hour after the sampling process, an observation attributed to the successful drug penetration into the skin, followed by subsequent release into the buffer medium.

To ensure the accuracy and reliability of the findings, the isolated tissue underwent an exhaustive and systematic examination conducted under strictly controlled conditions, effectively mitigating the risk of tissue degradation and contamination. Subsequently, the tissue functionality was assessed over 12 h timeframe, and to preserve its post-mortem state and enable examination of histo-toxicological alterations resulting from exposure to the membranes or buffers, the tissue was promptly fixed in formalin.

Consequently, a comprehensive histo-toxicological analyses have been carried out to offer a detailed and intricate characterization of the cellular and structural features of the tissue preparations. This check-point provided a profound understanding of the interactions between the membranes and the tissue at a microscopic level.

**Figure 6:**
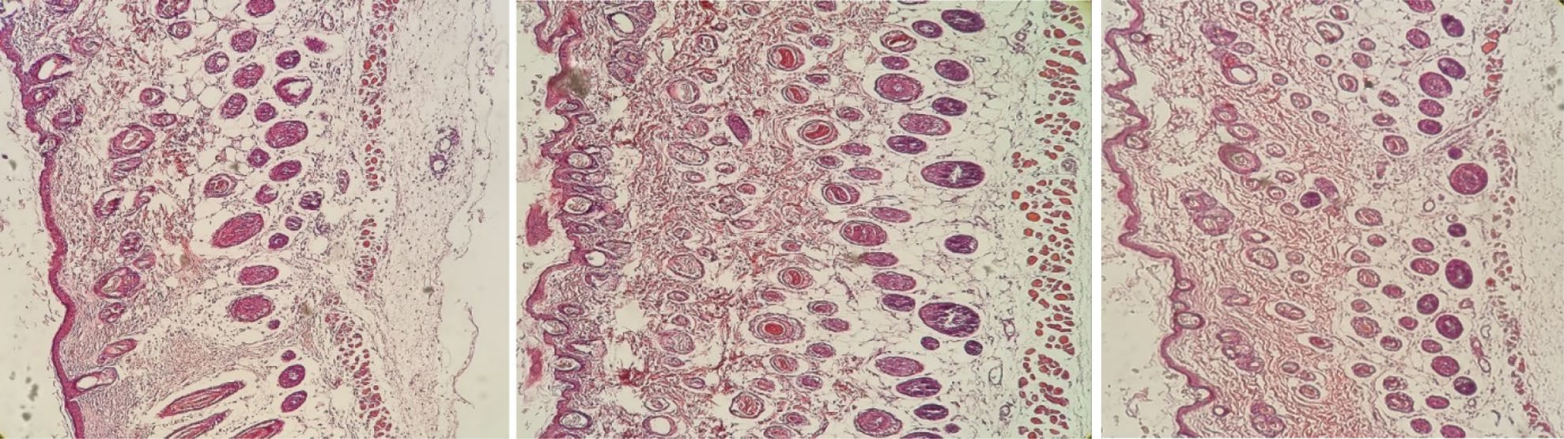
Comparison between M1 (left photo), M3 (middle photo), and M6 membrane (right photo) mouse dorsal skin assembly after 12 h of exposure to fabricated membrane (paraffin sections of mouse dorsal skin was stained with hematoxylin and eosin (10×)).

In conclusion, this investigation phase exemplified a systematic and methodologically refined approach to examining drug release behaviors by utilizing mouse dorsal skin samples and the M1 and M3 membranes. In this essence, we orchestrated procedures, along with controlled experimentation and exhaustive analysis, yielded precious insights into the distinct drug release patterns exhibited by the membranes and their consequential impact on the isolated tissue ex vivo **(**Table 4, Fig. 6**)**.

**Table 4:** The histo-toxicological evaluation of the mouse dorsal skin tissues after 8 h of exposure to fabricated membrane.

Membrane type	Keratin detachment	Epidermis integrity	ICSW*	Lipolysis	Subcutis integrity
M1	3.33 ± 0.236^a^	1.78 ± 0.147^a^	2.78 ± 0.147^a^	2.89 ± 0.261^a^	1.67 ± 0.167^a^
M3	2.33 ± 0.167^b^	1.89 ± 0.111^a^	2.33 ± 0.167^ab^	3.00 ± 0.289^a^	2.56 ± 0.176^b^
M6	1.56 ± 0.176^c^	3.22 ± 0.222^b^	2.00 ± 0.236^b^	2.11 ± 0.111^b^	3.00 ± 0.167^b^

The columns with different superscripts show significant differences between groups.

*ICSW intercellular space widening.

The keratin detachment has been varied among different membranes (F_2,24_ = 20.865; P_ANOVA_ = 0.000). In this line, keratin detachment has been significantly decreased in the SBA-15 modified membrane as compared to that of the bare membrane (P = 0.001). In addition, SBA-Q led to a significant reduction of keratin detachment compared to SBA-15 (P = 0.01) and bare membrane (P = 0.000). In this essence, we can conclude that the bare membrane may have more keratolytic activity than that of SBA-15 and SBA-Q modified membranes. The keratolytic activity of fabricated membranes may play a crucial role in the cutaneous penetrance of membrane-laden nanocompounds. The epidermis integrity has been varied among different membranes (F_2,24_ = 23.259; P_ANOVA_ = 0.000). The epidermis integrity of mouse skin tissues has been lost non-significantly in bare membrane rather than SBA-15 (P = 0.642) and significantly (P = 0.000) as compared with SBA-Q modified membrane, while Gln inclusion in SBA-Q showed a significant epidermoprotective activity in comparison to that of SBA-15 and bare membrane (P = 0.000). The hypodermis (subcutis) integrity has been changed among different membranes (F_2,24_ = 16.000; P_ANOVA_ = 0.000). In this continuum, hypodermis integrity has been lost significantly in bare membrane rather than SBA-15 (P = 0.001) and SBA-Q (P = 0.000) modified membranes.

The dermis dis-integrity is represented by increased ICSW and lipolysis. The ICSW (F_2,24_ = 4.353; P_ANOVA=_0.024) and lipolysis (F_2,24_ = 4.302; P_ANOVA=_0.025) have been altered among various membranes. The increased ICSW was more evident in bare and modified membranes. In this regard, ICSW has been expanded significantly in bare membrane rather than SBA-Q (P = 0.007) modified membrane. The lipolysis of the dermis layer was significantly lower in SBA-Q as compared to SBA-15 (P = 0.013) and bare membranes (P = 0.027). In sum, SBA-Q modified membrane conserved better the total architectural integrity of mouse dorsal skin tissues during diffusion investigation than the pristine bare membrane.

### Comparable evaluations

A comparison of activity properties for optimally modified PES membranes is reported in Table 5. The results indicated that modified membrane showed higher capability than the published data. As reported the M6 membrane exhibited desirable re-usable performance.

**Table 5:** Comparable evaluation of the obtained data and the literature review.

Drug name	Fabrication	Duration of release (h)	Antibacterial activity	Animal study	Ref.
Azithromycin	Nanoformulation	24	–	In vivo (mice)	^56^
Azithromycin	Niosomal gel	24	*Staphylococcus aureus Staphylococcus epidermidis*	Ex vivo (rat abdominal skin)	^57^
Vancomycin	Transferosome	24	–	Ex vivo (wistar rat skin)	^58^
Azithromycin	Hollow microsphere	12	–	In vivo (rabbit)	^59^
Azithromycin	Phase inversion	240	*Streptococcus sobrinus Escherichia coli Pseudomonas aeruginosa Staphylococcus aureus*	Ex vivo (mice skin)	This study

## Conclusion

This study revealed a new discovery for the development of a novel DDS using an asymmetric PES membrane integrated with SBA-15 and glutamine-modified mesoporous SBA-15 (SBA-Q) materials. The primary objective of the present study was to increase the effectiveness of AZI delivery for both in vitro and ex vivo applications. The secondary objective was to optimize the performance of the membrane according to critical parameters.

Furthermore, biocompatibility tests, including MTT assay, antibacterial activity analysis, blood compatibility evaluation, and histo-toxicological assessment, were conducted to assess the safety and biocompatibility of the modified membranes. The optimization identified an ideal membrane comprising 17% PES, 2% PVP, 1% SBA-15, and 0.5% SBA-Q. The optimized membrane exhibited a remarkable improvement in drug release compared to the unmodified membrane. This enhancement was attributed to the unique structural properties of the membrane, with the dense top layer facilitating sustained and continuous drug release. At the same time, the porous sub-layer acted as a drug reservoir. The drug release profile of the optimized membrane followed the zero-order model, and additional mathematical models were employed to investigate the underlying drug release mechanism.

Moreover, the biocompatibility assessments revealed promising biocompatibility of the optimized membranes, with cell viability of approximately 90% and minimal blood cell hemolysis (4.7%). Furthermore, long-term performance evaluation involving ten repeated usages demonstrated the reusability of the optimized membrane, highlighting its potential for sustained drug delivery.

### Supplementary Information

Supplementary Information.

## Data Availability

All data obtained during this experimental study are included in this published article and its Supplementary Information file.
