# Supplementary material for: Designing of a new transdermal antibiotic delivery polymeric membrane modified by functionalized SBA-15 mesoporous filler
Source: Sci Rep. 2024 May 6;14:10418. doi: 10.1038/s41598-024-60727-x (PMC11074140; doi:10.1038/s41598-024-60727-x)
Supplement: Supplementary file 1 — Supplementary Information. [file 41598_2024_60727_MOESM1_ESM.docx]

The objective of the study is to optimize the ratio of main polymer (PES) to pore maker (PVP) percentage for membrane fabrication. Different percentages of polymer and pore maker were evaluated (**Table raw supplementary (SR1)**). These variations were likely tested in a controlled laboratory setting. The performance of the membranes was assessed using microscopic analysis and functional tests. Specifically, hydrophilicity and porosity **(Table RS2)**, and drug release performance (**Fig. RS1**) were measured. After analyzing the data, the 17%PES and 2%PVP was identified as the optimal ratio for unmodified membranes. Modifications involving different percentages of SBA and modified SBA by glutenin were likely compared against this optimal ratio to assess any improvements or alterations in membrane properties.

| **Table RS1.** The composition of the fabricated membranes | | |
| --- | --- | --- |
|  | PES (%) | PVP (%) |
| 1 | 13 | 0 |
| 2 | 13 | 2 |
| 3 | 13 | 4 |
| 4 | 17 | 0 |
| 5 | 17 | 2 |
| 6 | 17 | 4 |
| 7 | 21 | 0 |
| 8 | 21 | 2 |
| 9 | 21 | 4 |

Note: PES: polyethersulfone; PVP: polyvinylpyrrolidone

| **Table RS2.** The membrane porosity and water contact angle of the fabricated membranes | | | | | | | | | |
| --- | --- | --- | --- | --- | --- | --- | --- | --- | --- |
| **Membrane type** | **13-0** | **13-2** | **13-4** | **17-0** | **17-2** | **17-4** | **21-0** | **21-2** | **21-4** |
| **Porosity (%)** | 16.1 | 44.12 | 43.79 | 10.67 | 41.52 | 45.11 | 9.68 | 37.46 | 38.45 |
| **WCA (º)** | 63.99 | 65.36 | 66.14 | 73.23 | 68.22 | 71.43 | 75.29 | 72.91 | 70.13 |

Note: WCA: water contact angle

| ****  **(a)** |
| --- |
| ****  **(b)** |
| ****  **(c)** |
| **Fig. RS1.** The release profile of the fabricated membrane, with 300µm thickness and 1500mg/L drug concentration |

**Characterization of SBA-15 and SBA-Q**

FTIR spectroscopy is an analytical technique to investigate the characteristics of the materials. The FTIR spectra of SBA-15 display distinctive peaks that offer insights into the chemical and physical properties (**Figure S1**). The Si-O-Si stretching vibration among these peaks was typically occurring at ̴1070 1/cm which indicates the presence of siloxane bonds within the SBA-15 structure, contributing to its stability and thermal resistance. Another peak arose from the Si-OH stretching vibration, commonly observed around 950 1/cm. This peak signified the existence of surface hydroxyl groups on the SBA-15 material, which is known to play a pivotal role in numerous catalytic and adsorption applications [1-4].

The prominent glutamine amide I band, typically observed in the 1600-1700 1/cm range, arose from the stretching vibration of the carbonyl (C=O) group in the amide (C=O-NH_2_) moiety. This band offers valuable information regarding the secondary structure of proteins containing glutamine. Another important band is the amide II, which appeared around 1500-1550 1/cm. It comprised a combination of N-H bending and C-N stretching vibrations within the amide group, providing further insights into the molecular composition.

Additionally, the amide III band, appearing in the 1200-1300 1/cm region, arose from a composite vibration involving N-H bending, C-N stretching, C-C stretching, and C-NH bending. This band contributed to interactions and conformational changes in glutamine-containing compounds' molecules. The carboxyl group typically generated an absorption peak around 1700-1750 1/cm, whereas the amino group involved in the spectrum had a distinctive peak in the 3300-3500 1/cm range.

| 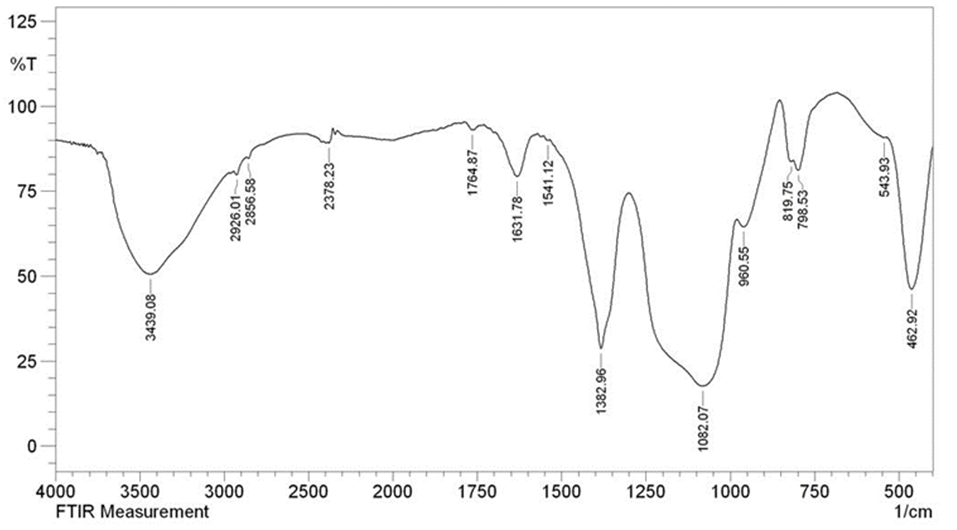  **SBA-15** |
| --- |
| 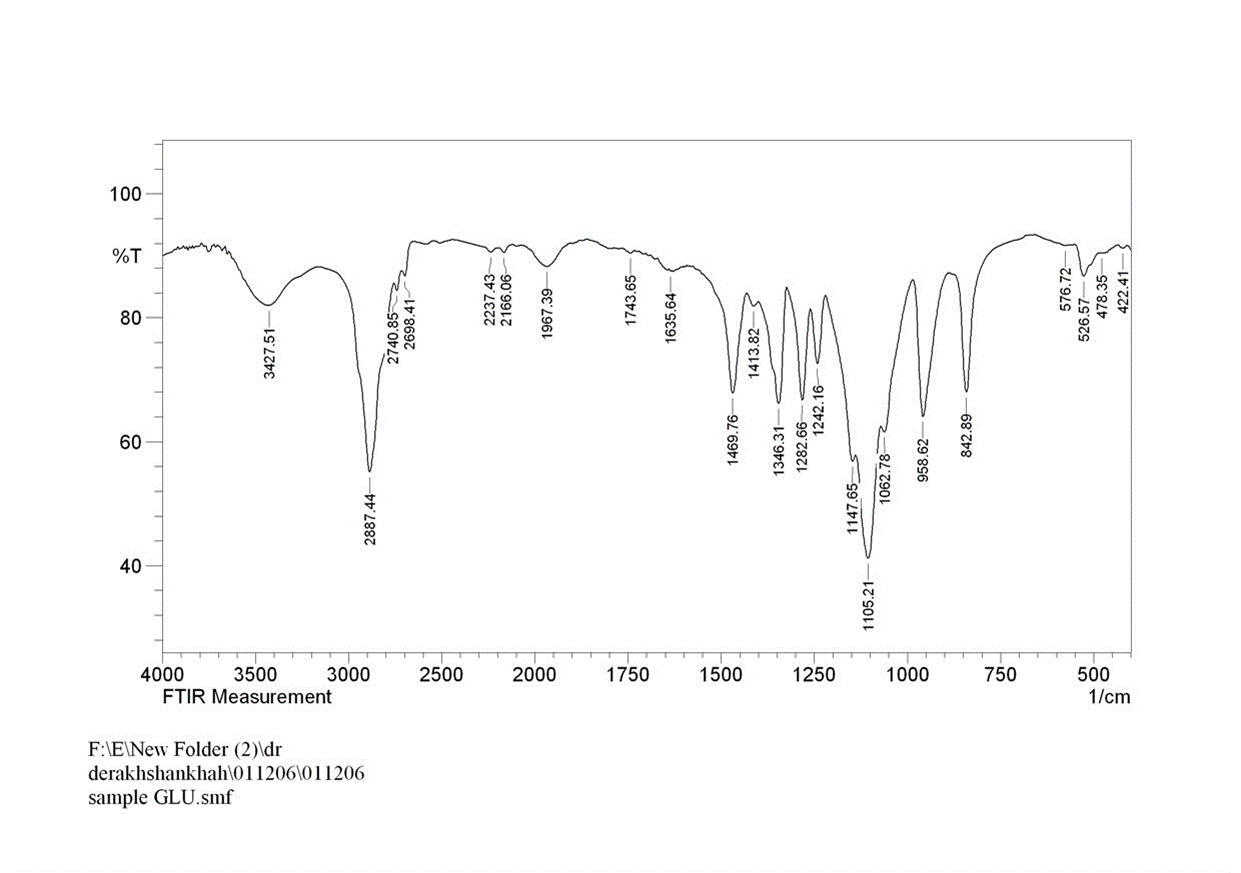  **Gln** |
| 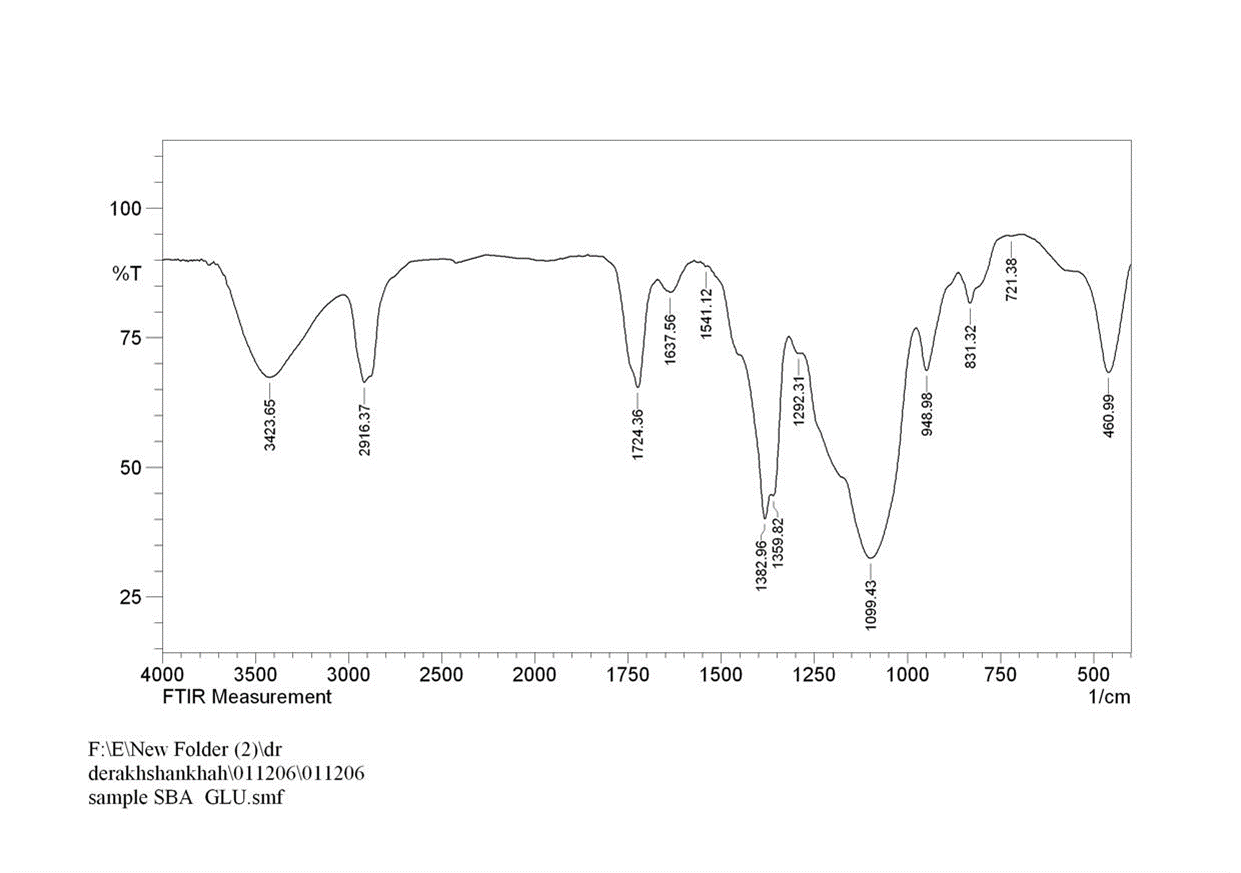  **SBA-Q** |
| **Figure S1.** The Fourier-transform infrared spectroscopy (FTIR) spectra of the SBA-15 and modified SBA-15 by glutamine (SBA-Q) |
|  |

**SEM images of the nanoparticles**

The SEM images were used to investigate structural characteristics and size distribution. Through examination of SBA-15, distinctive features can be obtained (**Figure S2**). The images exhibited a uniformity in size and shape, indicating the well-quality synthesis of SBA-15[5].

The surface of the SBA-Q material presented an almost uniform appearance, serving as a testament to the meticulous control by glutamine during the modification process. These observations validated the high caliber of the synthesis process, emphasizing the calm nature of the material formation.

| 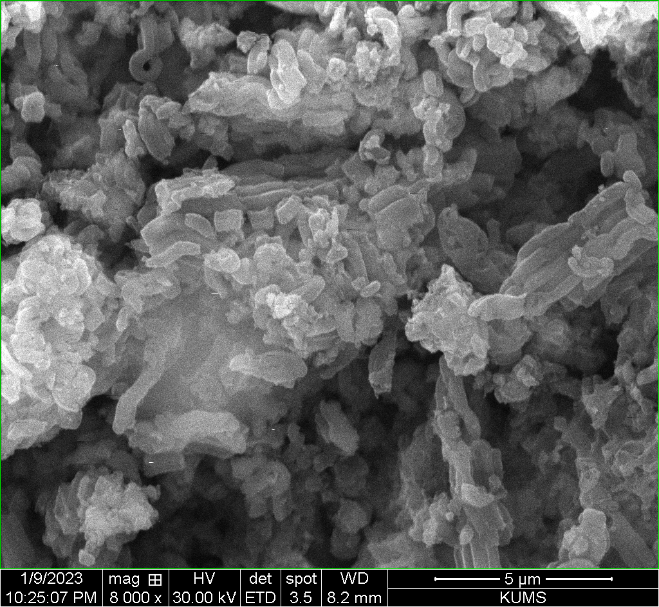 | 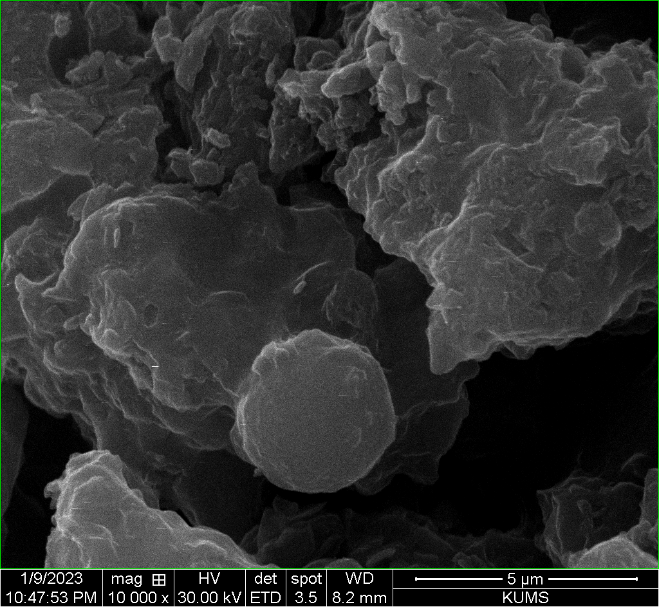 |
| --- | --- |
| SBA-15 | SBA-Q |
| **Figure S2.** The scanning electron microscopy image of the unmodified (SBA-15) and modified SBA by glutamine (SBA-Q) | |

|  |
| --- |
| **Figure S3.** The water contact angle (WCA) of the fabricated membranes (n=5) |

**Figure S4** shows the release profile of AZI (500 mg/L) from the fabricated membranes and provides insights into the release behavior.

The results showed that the optimal performance in AZI release was obtained when the membranes were modified with 1% SBA-15 and 0.5% SBA-Q concentration. Remarkably, the release of AZI reached 302.5 mg/L and 465 mg/L, respectively. This behavior can be attributed to the effect of optimal filler loading on the formation of large pores embedded in the membrane structure. The creation of these enlarged pores during the polymer film fabrication significantly contributed to efficient membrane modification and ultimately resulted in the maximum drug solution storage capacity.

In contrast, the unmodified membrane (M1) exhibited a burst release of AZI within the initial hours, followed by diminished performance over 24 hours, with a release of only 240 mg/L. This can be attributed to the absence of the optimized filler and, consequently, the lack of large pores necessary for efficient drug loading and release. The other modified membranes demonstrated smaller cavities, primarily due to suboptimal loading amounts of the mesoporous fillers. This reduction in cavity size consequently affected their drug absorption and release capacity, underscoring the significance of attaining the optimal loading concentration. These findings are important in selecting optimally modified membranes, specifically those loaded with 1%wt SBA-15 and 0.5%wt SBA-Q, for various applications where controlled drug release is critical.

Based on the drug release profiles obtained from previous experiments, it has been determined that the M3 and M6 membranes demonstrate the most effective drug release performance among both unmodified and modified membranes. Increasing the drug loading in the fabricated membranes leads to an increase in drug release. **Figure S5a and b** illustrates the drug release profiles for different initial drug loadings (500, 1000, and 1500 mg/L). Notably, the drug release rate exhibited an increasing trend with higher initial drug concentrations of 1000 and 1500 mg/L over 24 hours. Furthermore, the amount of drug released is influenced by various factors, including the physical and chemical properties of the membranes. For both the M3 and M6 membranes, the most favorable drug release profile was observed at an initial drug concentration of 1000 mg/L. This concentration displayed a higher rate of drug absorption and release from the membrane, resulting in a lower retention percentage than the other two concentrations (500 and 1500 mg/L).

The variation in drug release profiles between high and low drug concentrations can be attributed to the hydrophilicity of the membranes, which facilitates the formation of hydrogen bonds between the membrane matrix and the drug solution. This interaction can lead to incomplete drug release. Conversely, the lower drug concentration (1000 mg/L) exhibits a higher water content, leading to the formation of a molecular hydration layer on the surface and within the pores of the membrane. This hydration layer acts as a protective barrier, impeding drug accumulation and facilitating efficient drug transfer. This aspect of the study provides critical insights into the drug release behavior of modified membranes, highlighting the importance of optimizing both the membrane hydrophilicity and the drug concentration to achieve optimal drug release efficiency. By considering these parameters, it is possible to enhance the release kinetics and improve the overall performance of DDSs based on modified membranes.

**Figure S5,b** presents comparative data for the M1, M3, and M6 membranes at the optimal drug concentration (1000 mg/L). It is evident that the modified membranes exhibited a significant improvement in the drug release profile. This enhancement can be attributed to the optimal loading of fillers into the membrane matrix, which enhances the morphological (structural) and physical properties (hydrophilicity) of the membranes. Comparative analysis emphasizes the fundamental effect of membrane modification on drug release behavior. By incorporating nanoparticles at appropriate concentrations, the modified membranes show significant improvement in drug release ability. This increase can be attributed to the changes in the morphology of the membranes and the increase in their hydrophilicity.

The thickness of the membrane plays a critical role in determining the drug release profiles. To investigate this issue, the drug release was examined using optimally modified membranes (M3 and M6) at a concentration of 1000 mg/L in different membrane thicknesses (150, 300, 450, and 600 µm), as illustrated in **Figure S6**, the results revealed that the membranes with a thickness of 450 µm exhibited the optimal performance in drug release for both M3 and M6 membranes. This particular thickness demonstrated the best balance between drug-holding capacity and efficient drug release, making it the optimal thickness for the fabricated membranes in this study.

Initially, thicker membranes were anticipated to have a greater drug-holding capacity, leading to improved release profiles. However, increasing the membrane thickness decreased the drug release, as observed in the SEM images (**Figure 1**, 600 µm). The study unveiled that the increased thickness of the casting solution for membranes with a thickness of 600 µm influenced the kinetic and thermodynamic changes during the phase inversion process, thereby reducing the size of the membrane pores. This phenomenon can be attributed to the altered dynamics of the phase inversion process in thicker membranes, which decreased pore size and hindered drug release. This study elucidated the significant impact of membrane thickness on drug release profiles. Despite having a larger drug-holding capacity, thicker membranes exhibited reduced drug release. This can be attributed to the changes in the kinetic and thermodynamic factors altering during the phase inversion process, resulting in smaller membrane pores. These findings will underscore the importance of carefully considering and optimizing membrane thickness in DDSs to achieve desired drug release kinetics and efficiency.

|  |
| --- |
|  |
| **Figure S4.** *In vitro* drug release profiles of 500 mg/L AZI loaded in the M1– M9 in different filler loading (0.5, 1, 1.5, and 2%) with the Franz diffusion cell in the phosphate buffer solution medium pH 7.4 |

| a |
| --- |
|  |
| c |
| **Figure S5.** *In vitro*, drug release profiles in different drug concentrations (500, 1000, and 1500 mg/L of AZI) loaded in the M3 (a), M6 (b) (as the optimally modified membranes), and M1 (c) in optimal drug solution concentration (1000 mg/L) with the Franz diffusion cell in the phosphate buffer solution medium pH 7.4 |

|  |  |
| --- | --- |
|  |  |
| **Figure S6.** *In vitro* drug release profiles of the optimally modified membranes (M3 and M6) in different membrane thicknesses (150, 300, 450, and 600µm) |  |

|  |
| --- |
| **Figure S7.** The entrapment efficiency (EE) and drug loading (DL) of the M6 membrane in different drug feed concentrations (500, 1000, and 1500 mg/L, 450 µm) (n=3) |

|  |
| --- |
| **Figure S8.** The cell viability of the bare and optimally modified membranes, M1, M3, and M6, in 24, 48, and 72h (n=3) |

1. Moore, K.W., R. de Waal Malefyt, R.L. Coffman, and A. O'Garra, *Interleukin-10 and the interleukin-10 receptor.* Annu Rev Immunol, 2001. **19**: p. 683-765.

2. Wang, X., X. Du, H. Rao, and H. Chen, *The instant-direct-templating synthesis of phenyl-functionalized HOM-type mesoporous silica materials.* Applied surface science, 2009. **255**(12): p. 6073-6076.

3. Zhou, J., et al., *MnO2 nanosheet-assisted hydrothermal synthesis of β-MnO2 branchy structures.* Materials Letters, 2012. **79**: p. 288-291.

4. Moore, K., R. de Waal Malefyt, R. Coffman, and A. O'Garra, *Interleukin-10 and interleukin-10 receptor.* Annual Review of Immunology, 2001. **19**: p. 683-765.

5. Che, R., D. Gu, L. Shi, and D. Zhao, *Direct imaging of the layer-by-layer growth and rod-unit repairing defects of mesoporous silica SBA-15 by cryo-SEM.* Journal of Materials Chemistry, 2011. **21**(43): p. 17371-17381.
